# Identification of risk factors in epidemiologic study based on ROC curve and network

**DOI:** 10.1038/srep46655

**Published:** 2017-04-24

**Authors:** Jiao Jin, Shixin Zhou, Qiujin Xu, Jinbing An

**Affiliations:** 1 School of Statistics, Beijing Normal University, Beijing, 100875, China; 2 Department of Cell Biology, School of Basic Medicine, Peking University Health Science Center, Beijing, 100191, China; 3 Chinese Research Academy of Environmental Science, Beijing, 100012, China; 4 Faculty of Foundational Education, Peking University Health Science Center, Beijing, 100191, China

## Abstract

This article proposes a new non-parametric approach for identification of risk factors and their correlations in epidemiologic study, in which investigation data may have high variations because of individual differences or correlated risk factors. First, based on classification information of high or low disease incidence, we estimate Receptor Operating Characteristic (ROC) curve of each risk factor. Then, through the difference between ROC curve of each factor and diagonal, we evaluate and screen for the important risk factors. In addition, based on the difference of ROC curves corresponding to any pair of factors, we define a new type of correlation matrix to measure their correlations with disease, and then use this matrix as adjacency matrix to construct a network as a visualization tool for exploring the structure among factors, which can be used to direct further studies. Finally, these methods are applied to analysis on water pollutants and gastrointestinal tumor, and analysis on gene expression data in tumor and normal colon tissue samples.

Identification of possible risk factors of specific diseases in epidemiologic studies is helpful in guiding diagnosis, therapy or disease control. This process is usually considered as a problem of variable selection in mathematics. However, due to individual differences or complicated interaction of risk factors, the epidemiologic investigation data often have serious variation and the relationship between response variable and explanatory variables can not be appropriately expressed by specific mathematical models, which may reduce the reliability of classical methods for variable selection. Therefore, it is desirable to develop appropriate analysis methods suitable for the epidemiologic data.

The conventional methods for variable selection include steps to construct some evaluation functions based on specific parametric models and identify significant risk factors through optimization process^1^,^2^. These methods usually have severe limitations on the distribution of random errors and mathematical forms of models, such as linear model^3^, Cox model^4^,^5^ and logistic model^6^. However, besides influence of large variation of observations, the bias of selected mathematical model may lead to inappropriate conclusions^7^,^8^. For example, some important variables may be rejected by selected model mistakenly, or inconsistent conclusions may be obtained after use of different models.

In contrast to parametric methods, random forest is often used to select variables through change of certain measurement on prediction accuracy when selected variables are eliminated^9^,^10^,^11^. In addition, methods based on some probability function^12^,^13^ or network^14^,^15^ are also effective choices to evaluate specific genes or tissues in studies of biomedical science. These methods are non-parametric methods without severe limitations on models or data, and therefore more suitable for the problems with high variation data and unknown factor structure in epidemiologic studies.

Noting the binary feature of high and low disease incidences in epidemiologic investigation data, and two components of true positive rate (TPR) and false positive rate (FPR) in ROC curve^16^,^17^, we select ROC curve to describe the relationship between risk factors and disease incidence, and screen for the candidate important risk factors. ROC curve has a well-established theoretical basis^18^,^19^, and is widely used for many problems^20^,^21^. Furthermore, we define a new type of correlation matrix based on distance of ROC curves corresponding to any pair of factors, and then use it to evaluate the correlated effect of risk factors on disease and to construct a network as a visualization tool for exploring the structure among factors.

## Screening of risk factors based on ROC curve

Suppose that k-dimensional random vector 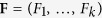 denotes the risk factors, where each 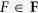 has nonempty support set 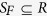, and random variable *D* denotes the state of disease, where *D* = 1 represents diseased population, and *D* = 0 represents healthy population. To study the impact of **F** on disease incidence *π*, we can investigate observation 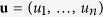 from diseased population, and 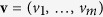 from healthy population, where each vector *u*_*i*_ or *v*_*j*_ denotes observations of factors 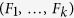.

For any factor 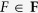, ROC curve is defined as a graph of true positive rate (TPR) in y-axis versus false positive rate (FPR) in x-axis. For the sake of simplicity, ROC can be expressed by a series of (*R*_*X*_(*f*), *R*_*Y*_(*f*)) in coordinate system *X* × *Y* for various values of 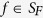, where


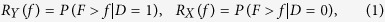


and their values can be estimated by **u** and **v**, respectively.

Because both *R*_*X*_(*f*) and *R*_*Y*_(*f*) are monotone functions with range (0,1) and connected by common value 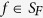, if we define 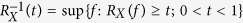, then ROC curve can also be expressed as a graph of (*t, R(t*)) with only one parameter *t* on (0, 1):


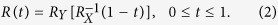


Now, suppose the larger value of the variable *F* increases the disease incidence *π*, that is for 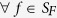, 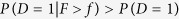, then according to definition of conditional probability, we can reach the conclusion:


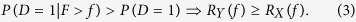


Similarly, for 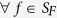, we can also obtain


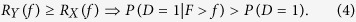


Because of the equivalence property of (*R*_*X*_(*f*), *R*_*Y*_(*f*)) and (*t, R(t*)), the conclusions above suggest that if the larger value of the variable *F* increases the disease incidence *π*, then the ROC curve *R(t*) is above the diagonal of bounded region 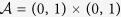 constantly. Similarly, if *R(t*) is above the diagonal of region 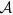 constantly, then the larger value of the variable *F* may result in the larger disease incidence *π*.

Based on this fact, we can evaluate whether *F* plays an important role in influencing disease incidence *π* through hypothesis testing with null-hypothesis of independence between variable *F* and *D*, that is 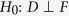. To construct appropriate test statistic, suppose consistent estimation of *R(t*) is 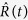, then using the conclusion on ROC curve^22^, as 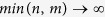, *n/m* → *λ*, we have


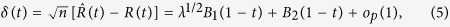


where *B*_1_(*t*) and *B*_2_(*t*) are two identical independent Brownian bridges.

Suppose the null hypothesis of *H*_0_ is true, we should have 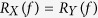 for 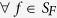, which means that *R(t*) = *t*. In this situation, if we define the symbol 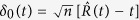, then we can obtain its asymptotic distribution based on Brownian bridge. Because the integral on *δ*_0_(*t*) is connected with Area under ROC Curve (AUC), which is well known in epidemiologic study, we can construct test statistic based on AUC:


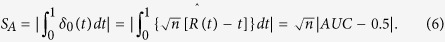


If the value *S*_*A*_ is larger than a certain critical value, we can reject the null hypothesis of *H*_0_, which means that the impact of variable *F* on disease incidence *π* can not be explained as random fluctuations.

Because the asymptotic distribution of *δ*_0_(*t*) is expressed as a linear combination of two Brownian bridge processes, we can obtain the empirical distribution of test statistic *S*_*A*_ through method of asymptotic simulation, and judge whether the ROC curve is significantly deviated from the diagonal. Specifically, we can simulate two independent Brownian bridges *B*_1_(*t*) and *B*_2_(*t*) using relationship between Brownian bridge and Brownian motion, and construct stochastic process of *δ*_0_(*t*) by equation (5) and *S*_*A*_ by equation (6). Repeat this process for *n* times, and we can obtain *n* simulated observations of *S*_*A*_, from which we can obtain the empirical distribution of test statistic *S*_*A*_ together with the hypothesis threshold as the null hypothesis *H*_0_ is true, and then we can complete the hypothesis test to judge whether the ROC curve has significant deviation from the diagonal, which can be used to screen for the variable 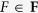 with important impact on disease.

## Construction of network based on correlation matrix

Although ROC curve can express the effects of any variable 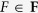 on disease *D*, we can only evaluate their effects one by one. In fact, the variables often have correlation with one another, therefore it is necessary to analyze the interaction among risk factors. Similarly, the measurements of correlation, such as Pearson coefficient of correlation, Kendall coefficient or other measurements, also have such disadvantages, which can not express the correlation among many variables.

Considering the unknown structure of correlation among many variables, we select network to evaluate their correlation. Network is constructed by many knots with connections between certain pair of variables and therefore can describe the complex interaction among interesting variables^23^,^24^.

Provided that two factors *F*_*i*_ and *F*_*j*_ have synergistic effect, they should have similar ROC curves and small value of difference between their AUC. Based on the assumption above, we define distance *d*_*ij*_ between any pair of variables of *F*_*i*_ and *F*_*j*_ to evaluate the correlation of *F*_*i*_ and *F*_*j*_ on disease *D*:


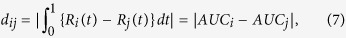


whose value can be estimated by 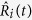 and 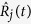, and we can denote its estimation as 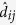. Then, we select the expression below to estimate the correlation of *F*_*i*_ and *F*_*j*_:


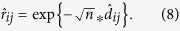


Here, the value of 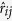 is always confined into interval of (0, 1), and the larger value of 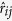 is, the stronger correlation of *F*_*i*_ and *F*_*j*_ in effects on disease *D* should be.

If we obtain all the estimations of correlations among factors 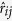, then we can use the matrix of 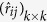 as adjacency matrix to construct network to analyze the structure of correlation among these factors. However, when the value of *k* × *k* is too large, some connections may be noisy ones, whose values should be converted into 0 through some criteria to avoid interference of them on the analysis.

In fact, based on equation (5), for any pair of *F*_*i*_ and *F*_*j*_, we have


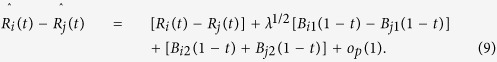


Based on this result, suppose *F*_*i*_ and *F*_*j*_ have strong connection and similar ROC curves, and express it as null hypothesis *R*_*i*_(*t*) = *R*_*j*_(*t*), then the value of 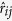, as a function of 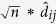, can also be evaluated by the aid of distribution of Brownian bridge. Thus, it is a good choice to make decision on whether certain connections 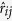 should take values 0 through approximated distribution of 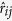, just as some statistical methods do in testing whether some parameters should take values 0.

Now, similar to the process of simulation mentioned above, we can simulate Brownian bridges *B*_*i*1_, *B*_*i*2_ and *B*_*j*1_, *B*_*j*2_, from which we can obtain simulated values of 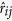 by equations (7) and (8). Repeat this procedure for *n* times, and we can obtain *n* simulated observations of *r*_*ij*_ and obtain its empirical distribution. Then, for a given level *β*, we take the quantile of empirical distribution as threshold and transform the ones lower than threshold to 0. Here, the smaller the value of *β* is, the fewer nodes in the network there are.

After rearrangement of each element, the matrix 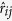 should have more explicit information. Therefore, we can take the amendatory matrix 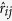 to construct a network to explore the relationship among these factors, such as individual groups and their central nodes, which can be clues for further experimental or theoretical studies.

The methods above are completed by R software, and the programs are available in appendix, through which the readers can update the programs based on their new methodology.

## Examples and Results

### Example 1

In this part, we apply the introduced methods to problem of correlation between gastrointestinal (GI) tumors and pollutants in local drinking water, particularly polycyclic aromatic hydrocarbons (PAHs) and heavy metals. Some reports have suggested that high levels of PAHs in the air may be associated with cancer^25^,^26^,^27^. However, few studies have assessed the presence of both PAHs and heavy metals in sources of drinking water, which may have stronger influence in GI tumors.

In the current study, Huai’an region, located in the middle of Jiangsu Province, has been one of the surveillance spots with high cancer incidence for 30 years in China. Furthermore, Huai’an has the highest incidence of GI cancers in Jiangsu Province, and patients suffering from GI cancers (mainly esophageal, stomach, and liver cancers) account for more than two-thirds of all cancer patients in Huai’an^28^. Therefore, on the basis of the cancer surveillance data for incidence and mortality, three counties in Huai’an (Xuyi, Jinhu, and Chuzhou) with a high cancer incidence were selected as test group, and the Tongshan district of Xuzhou city, which has a low cancer incidence, was selected as the control group.

To study the important risk factors which may affect the disease incidence *π*, based on related literature and other information source about candidate risk factors of GI tumors, we select and measure 25 risk factors for each sample of water, including 15 PAHs and 10 heavy metals, and the *jth* factor is denoted as *F*_*j*_, whose observations are denoted as *f*_*ij*_.

Firstly, we give the basic information of all the 25 risk factors by two box graphs, where one is corresponding to test group and the other to control group. However, considering that observations correspond to different substances, which suggest that the values may not be comparable to each other, and each variable may have high variability, we make data transformation on raw data by monotone function below


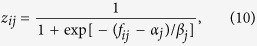


where the parameters of *α*_*j*_ are median of *f*_*ij*_, and *β*_*j*_ are median absolute deviation of *f*_*ij*_. Through data transformation on raw data, all the observations can be confined into interval of (0, 1), which can ensure that the data of each variable is comparable in a single box graph. In fact, this transformation is not necessary for analysis in this article, because monotone transformation will not change the values of *R*_*X*_(*t*) and *R*_*Y*_(*t*), therefore the ROC should be same to former ones.

**Figure 1:**
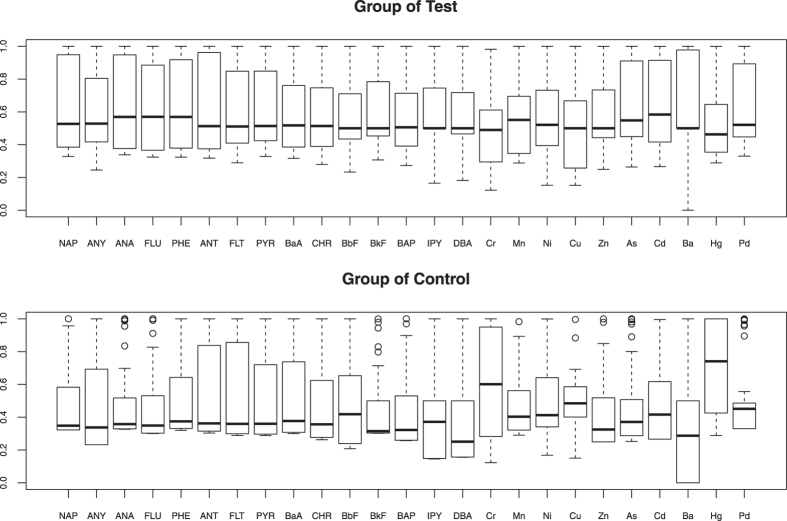
Box graphs of 25 candidate risk factors.

The box graphs on transformation *z*_*ij*_ are shown in Fig. 1. According to this figure, the values of many factors have high variation, which means that it is not very reliable to perform conventional statistical analysis based on such investigation data. For example, we perform variable selection by logistic model for each variable at one time, and only 5 variables are selected at level *α* = 0.05: BkF (V12), Cr (V16), Zn (V20), As (V21) and Ba (V23). Through probit model, we obtain similar results.

Secondly, we classify the samples with high and low cancer incidences as the test group and the control group, which are denoted as D = 1 and D = 0, respectively. And then, for each *F*_*j*_ from 25 risk factors, based on the information of 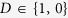, we calculate its ROC curve *R*_*j*_(*t*) and obtain the value of test statistic *S*_*Aj*_ based on equation (6). Then, through process of simulation for *n* = 1,000 times, we obtain the empirical distribution of test statistic *S*_*A*_ under null hypothesis *H*_0_, and then make hypothesis testing and give p-value of each observation of *S*_*Aj*_. The p-values are shown in Table 1, and Cu (V19) is excluded from candidates at level *α* = 0.05.

Furthermore, we also carry out variable selection by random forest based on the R package ‘*randomForest*’ for comparison. The parameter of ‘*ntree*’ is 1,000, and the measurement of importance for variables is ‘*Accuracy*’. We also provide the top 10 variables: BkF (V12), ANY (V2), NAP (V1), Hg (V24), IPY (V14), FLU (V4), BAP (V13), PYR (V8), ANT (V6) and DBA (V15). However, if the measurement of importance for variables is changed to ‘*Gini*’, then the top 10 variables are: BkF (V12), ANY (V2), NAP (V1), FLU (V4), FLT (V7), Cr (V16), ANA (V3), PYR (V8), BAP (V13) and CHR (V10). These results show that methods based on ROC and random forest, as nonparametric methods, give close conclusions, and the results are accorded with experimental study, which implies good performance of nonparametric methods.

**Figure 2:**
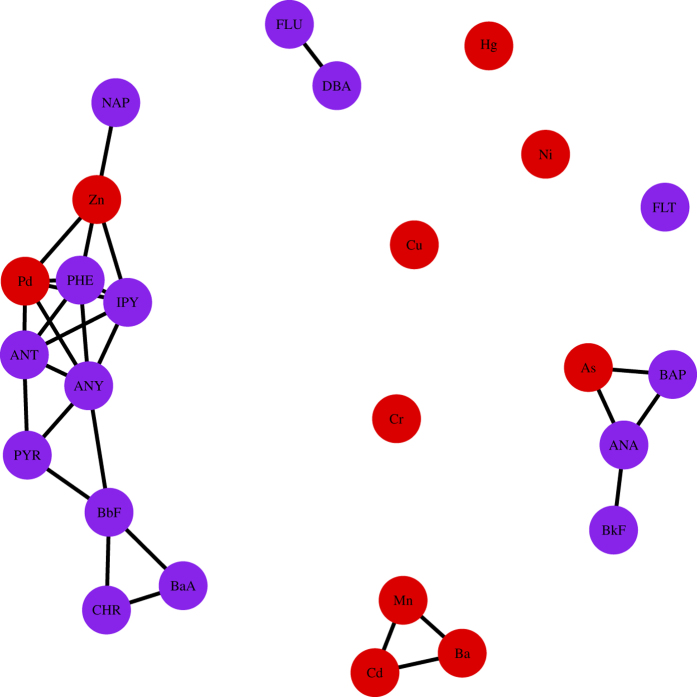
Network of water pollutant risk factors based on 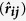 as *β* = 0.01.

**Figure 3:**
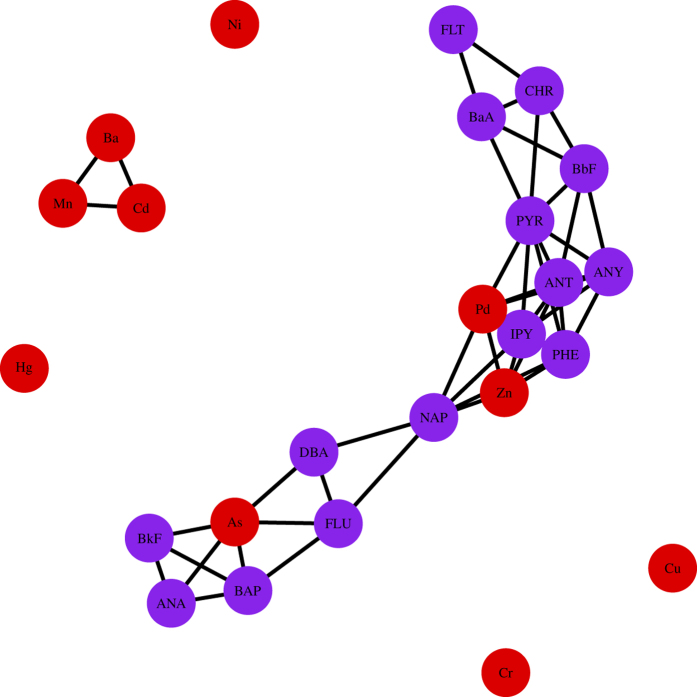
Network of water pollutant risk factors based on 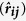 as *β* = 0.02.

Finally, we obtain measurement 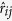 between any pair of variables *F*_*i*_ and *F*_*j*_ based on equation (8) and obtain matrix of 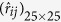, wherein the values lower than threshold at given level *β* are converted into 0. Then, based on the R package ‘igraph’, we take 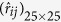 as adjacency matrix to construct network, where each node *V*_*k*_ corresponds to certain factor *F*_*k*_, and the purple dots and red dots denote the PAHs and heavy metals, respectively. The networks are shown in Figs 2 and 3, where the level *β* take values of 0.01 and 0.02, respectively.

Based on analysis of network, we can see that almost all the PAHs act as a group, and these results match the studies of PAHs and heavy metals for environmental pollution, such as air pollution, and cancer development^25^,^29^,^30^,^31^. In particular, we find that heavy metal As (V21) has strong connection with most PAHs. This finding of connection between As (V21) and PAHs may imply the existence of PAHs-arsenic co-contaminated sites^32^, because many PAHs-arsenic co-contaminated sites, such as wood preservation sites, coking or chemical industry sites, and mining or metallurgy industry sites, are common around our survey locations. This finding may indicate the importance of remediation technologies for PAHs-arsenic combined pollution in the future, such as microbial degradation methods^33^,^34^,^35^.

**Figure 4:**
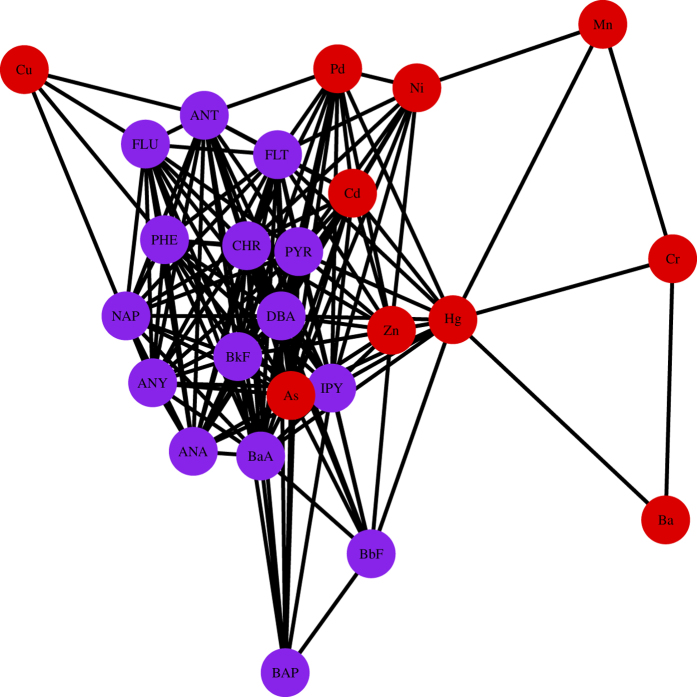
Network of water pollutant risk factors based on 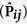 as *β* = 0.01.

For comparison, we also use Pearson sample correlation coefficient matrix 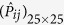 as adjacency matrix to construct network. Similar to the process on matrix 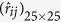, the ones lower than the threshold through test hypothesis on coefficient are converted into 0, and the network based on 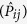 as *β* = 0.01 is shown in Fig. 4. We can find that this network can hardly give more information, and this phenomenon may be resulted from the sensitivity of *P*_*ij*_ on outliers of observation and the nonlinear relationship between some pairs of variables of *F*_*i*_ and *F*_*j*_.

### Example 2

To show more application of this method, we also use it to analysis of gene expression data in colon tissues, where the data is produced by U. Alon (1999). In this data set, the gene expression in 40 tumor and 22 normal colon tissue samples was analyzed with an Affymetrix oligonucleotide array complementary to more than 6,500 human genes, and two thousand out of around 6,500 genes were selected based on the confidence in the measured expression levels^36^.

In this example, we consider the genes in this data set as risk factors, and obtain about 100 genes as *β* = 0.01. Through the annotations of these candidate genes, we note that there are some genes having function connected with tumor of colon. For example, cadherins are the principal components of Adhesion Junctions (AJs) and cluster at sites of cell-cell contact in most solid tissues. These cell adhesion molecules play a significant role in the development of colorectal cancer and mediate the metastases of this common malignancy. Loss or downregulation of E-cadherin expression is a significant feature for colorectal cancer progression or the development of metastases^37^,^38^. Furthermore, besides E-cadherin, some other genes involved in the signaling pathway of Adhesion Junctions (AJs), including LAR protein, DEP1 (Protein Tyrosine Phosphatase), alpha-catenin, alpha-actinin and actin, also appear in this candidate gene set. These genes together with their annotations are shown in Table 2. The fact that quite a few genes in Adhesion Junctions coexist in the filtered gene set indicates this method can be used to screen for the genes related to colorectal cancer.

**Figure 5:**
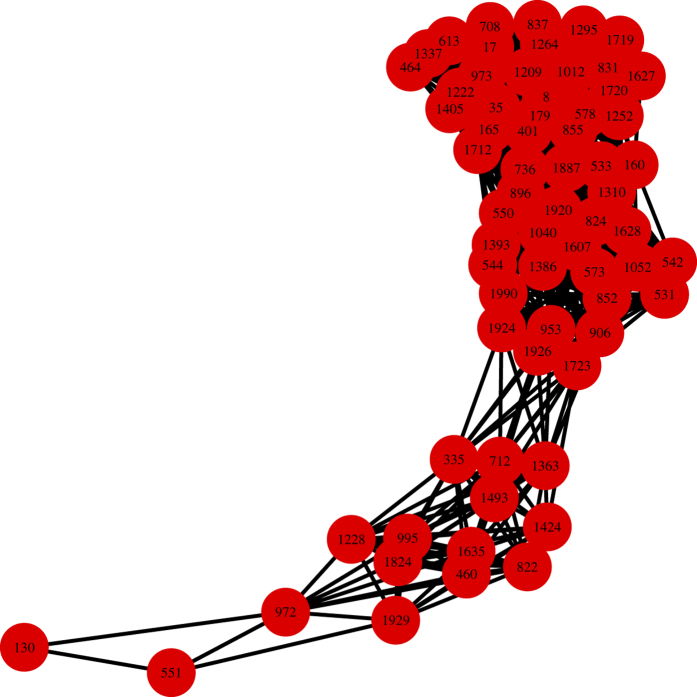
Network of genes in colon data based on 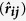 as *β* = 0.01.

We also construct network based on 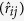 corresponding to these candidate genes as level *β* = 0.01 and the result is shown in Fig. 5, through which we find that these genes can be roughly divided into two groups, where the six genes except LAR protein coexist in one group, and LAR protein is in the other group. It suggests that our method may give clues to connections among genes.

The data sets used in Example 1 are presented in files of “Supplementary Data 1.csv” and “Supplementary Data 2.csv”, which are the observations and classification information of samples, respectively. The data set used in Example 2 is produced by U. Alon (1999) and is available on the web at http://www.molbio.princeton.edu/colondata. The programs for data analysis in Example 1 and Example 2 are presented in “Supplementary RiskFactor.R”.

## Discussion

In epidemiologic studies, because of high variability, complex structure among correlative factors, and individual differences of data, it is unreasonable to construct specific mathematical models directly to study the influence of risk factors on disease, while the proposed methods, as non-parametric statistical methods without severe mathematical conditions, such as normality or linear style as in classical statistical methods, are appropriate to explore the relationship between various risk factors and disease incidence. Specifically, ROC curve is only related with probability functions *R*_*X*_(*t*) and *R*_*Y*_(*t*), and can be estimated directly by quantiles, thus the statistic *S*_*A*_ or 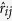 based on ROC curve is not sensitive to outliers, variability of data or individual differences, and can give more reliable conclusions.

Furthermore, although there is no explicit formulation between risk factors and disease incidence, according to equations (3) and (4), ROC curves imply the dose-effect relationship between selected risk factors and disease incidence, similar to classical linear model, which should help to evaluate and screen for the important candidate risk factors. Admittedly, because the larger values of the variable *F* do not necessarily increase or decrease the disease incidence *π* directly, this method may miss some factors without clear dose-effect relationship between *F* and *π*, therefore the users should pay attention to such limitation in real work.

In addition, as factors have complex correlation with each other, network analysis is a desirable choice to explore the complex interaction among different factors through many pairs of factors. The proposed method gives a nice visualization of the network based on correlation matrix *r*_*ij*_ among all risk factors. It is worth noting that the definition of *r*_*ij*_ is constructed by *R*_*i*_(*t*) and *R*_*j*_(*t*), which uses the information both from risk factors and disease status, while the traditional correlation matrix only uses the information from risk factors. Thus, this method can give more important information in exploring complicated relationship between risk factors and the disease in epidemiologic studies, and is helpful for directing further experimental analyses.

Finally, as shown in the two examples, the proposed method may provide useful tools in other biomedical problems with similar data structure. We can screen risk factors and filter for certain connections *r*_*ij*_ in network by relatively objective criterion, namely quantile of distribution, which can be approximated by some function of Brownian bridge. Thus, it is desirable in real studies, especially for the problems with big data, where some criterions, such as number of selected objects or proportion of total candidates, may be inconvenient for further studies. Incidentally, because the obtained networks may be too complex to efficiently interpret, it is still necessary to improve the proposed method to simplify the networks more efficiently and reliably in the future.

## Additional Information

**How to cite this article:** Jin, J. *et al*. Identification of risk factors in epidemiologic study based on ROC curve and network. *Sci. Rep.* **7**, 46655; doi: 10.1038/srep46655 (2017).

**Publisher's note:** Springer Nature remains neutral with regard to jurisdictional claims in published maps and institutional affiliations.

## Supplementary Material

Supplementary Information

Supplementary Dataset 1

Supplementary Dataset 2

## Figures and Tables

**Table 1:** P-Values corresponding to different water pollutant risk factors.

NAP(V1) 0.000	ANY(V2) 0.000	ANA(V3) 0.000	FLU(V4) 0.000	PHE(V5) 0.000
ANT(V6) 0.000	FLT(V7) 0.0040	PYR(V8) 0.000	BaA(V9) 0.004	CHR(V10) 0.004
BbF(V11) 0.004	BkF(V12) 0.000	BAP(V13) 0.000	IPY(V14) 0.000	DBA(V15) 0.000
Cr(V16) 0.012	Mn(V17) 0.048	Ni(V18) 0.104	Cu(V19) 0.832	Zn(V20) 0.000
As(V21) 0.000	Cd(V22) 0.004	Ba(V23) 0.000	Hg(V24) 0.000	Pd(V25) 0.000

**Table 2:** Annotations for some genes in signaling pathway of Adhesion Junctions.

ID	Name	Description
481	R09468	PROTEIN-TYROSINE PHOSPHATASE PTP-S (Rattus norvegicus)
806	Z13009	H. sapiens mRNA for E-cadherin
1337	R70016	Human F-actin capping protein beta subunit mRNA, complete cds
1393	X55187	Human mRNA for alpha-actinin, partial cds
1720	U03100	Human alpha2(E)-catenin mRNA, complete cds
1929	Y00815	Human mRNA for LCA-homolog. LAR protein (leukocyte antigen related)
